# Hydrogen Sulfide Plays an Important Role in Diabetic Cardiomyopathy

**DOI:** 10.3389/fcell.2021.627336

**Published:** 2021-02-18

**Authors:** Shizhen Zhao, Xiaotian Li, Xinping Li, Xiaoyun Wei, Honggang Wang

**Affiliations:** Institute of Biomedical Informatics, Bioinformatics Center, School of Basic Medical Sciences, Henan University, Kaifeng, China

**Keywords:** hydrogen sulfide, diabetic, cardiovascular diseases, diabetic cardiomyopathy, diabetic vascular diseases

## Abstract

Diabetic cardiomyopathy is an important complication of diabetes mellitus and the main cause of diabetes death. Diabetic cardiomyopathy is related with many factors, such as hyperglycemia, lipid accumulation, oxidative stress, myocarditis, and apoptosis. Hydrogen sulfide (H_2_S) is a newly discovered signal molecule, which plays an important role in many physiological and pathological processes. Recent studies have shown that H_2_S is involved in improving diabetic cardiomyopathy, but its mechanism has not been fully elucidated. This review summarizes the research on the roles and mechanisms of H_2_S in diabetic cardiomyopathy in recent years to provide the basis for in-depth research in the future.

## Introduction

Diabetes has reached epidemic levels, and its prevalence has increased significantly in recent years. In the 1990s, the number of people with diabetes worldwide was ~135 million, and this figure could reach 300 million by 2025. Cardiomyopathy is an important complication of diabetes, and more than 50% of diabetic patients die of cardiomyopathy (Balakumar et al., 2016; Htay et al., 2019). Diabetic cardiomyopathy (DCM) is a disease that leads to myocardial dysfunction without major risk factors (including hypertension or coronary artery disease) (Jia et al., 2018b). DCM is characterized by various forms, including hypertrophy of cardiomyocytes, reduction of myofibrils, interstitial fibrosis, and myocardial microvascular lesions (Russo and Frangogiannis, 2016; Varma et al., 2018). It has been reported that hyperglycemia, lipid accumulation, oxidative stress, inflammation, calcium overload, mitochondrial dysfunction, myocarditis, and apoptosis might be related with the pathogenesis of DCM (Jia et al., 2018a; Dillmann, 2019). However, the mechanism of DCM has not been fully studied. Therefore, it is particularly important to explore the pathogenesis and treatments of diabetic cardiovascular diseases. It has been reported that hydrogen sulfide (H_2_S) has many biological functions (Sun H. J. et al., 2019) and is involved in many diseases (Wang et al., 2020a), including diabetes-related diseases (Qian et al., 2018). This review summarizes the research about the roles and mechanisms of H_2_S in DCM in recent years to provide the basis for in-depth research in the future.

## Overview of DCM

The term *diabetic cardiomyopathy* was first used by Rubler in 1972. DCM increased the risk of heart failure in diabetes by four to five times. It is characterized by myocardial cell loss and myocardial fibrosis, ventricular systolic and/or diastolic dysfunction, without significant coronary atherosclerosis and hypertension (Poornima et al., 2006; Karbasforooshan and Karimi, 2017; Yao et al., 2018). In the early stage of DCM, the pathological alterations are mainly in the myocardial interstitium, including the formation of non-enzymatic advanced glycation end products (AGEs), impaired compliance, and ischemic intravascular diseases. Although the morphology of myocardial cells and small coronary arteries has been preserved anatomically, these changes still lead to impaired myocardial contractility. With the development of the disease, left ventricular (LV) hypertrophy is characterized by hypertrophy of myocardial cells, interstitial fibrosis and perivascular fibrosis, thickening of the capillary basement membrane, and formation of intracapillary microaneurysms (Felicio et al., 2016; Jia et al., 2018a). DCM occurs in both type 1 and type 2 diabetes mellitus (Sulaiman et al., 2010), affects nearly two-thirds of diabetic patients, and is the leading cause of diabetes-related morbidity and mortality (Boudina and Abel, 2007; Tillquist and Maddox, 2012). Although the strict control of blood glucose seems to play an important role in the prevention and treatment of DCM, there is an urgent need for new methods and specific therapeutic agents for DCM. Therefore, it is necessary to understand the pathogenesis of DCM (Acar et al., 2011). The molecular mechanism of DCM may be multifactorial, including but not limited to inflammation, cell death, nitroso stress, oxidative stress, impairment in calcium handling, increased AGEs, and mitochondrial dysfunction (Al Hroob et al., 2019). These pathologic alterations in cardiomyocytes are primarily triggered by many metabolic disorders including hyperglycemia, dyslipidemia, increased free fatty acid (FFA) release, and insulin resistance (Mahmoud, 2017). However, the pathogenesis of DCM remains to be elucidated.

## Overview of H_2_S

**Figure 1:**
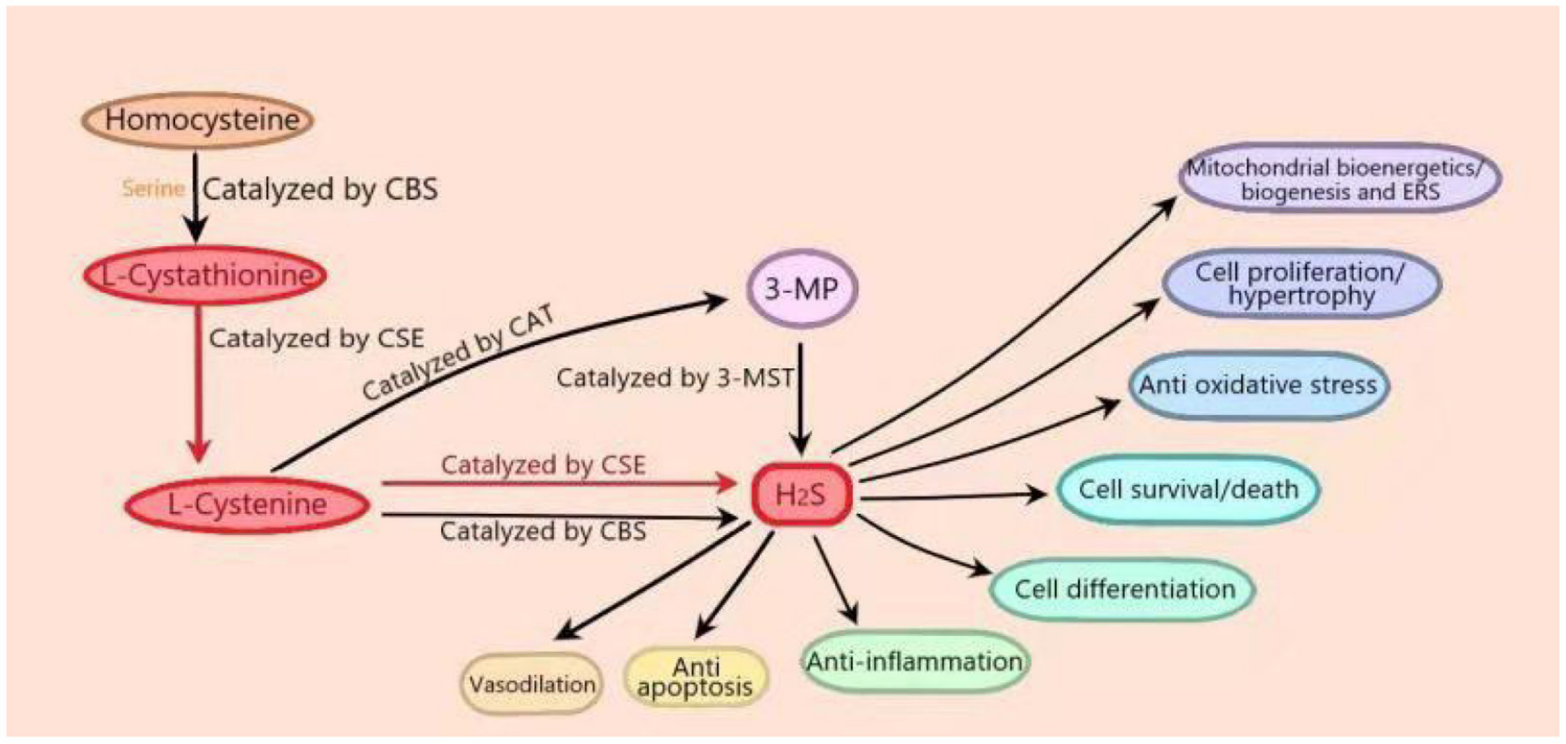
*In vivo* synthesis process and biological function of hydrogen sulfide (H_2_S). In the process of endogenous H_2_S production, firstly, cystathionine-β-synthase (CBS) catalyzes the β-substitution reaction of homocysteine with serine to produce L-cystathionine. L-cystenine is produced by the elimination of α, γ-cysteine of L-cystathionine catalyzed by cystathionine-γ-lyase (CSE). L-cystenine then produces hydrogen sulfide (H_2_S) via β elimination reaction catalyzed by CSE/CBS. L-cystenine also produces 3-mercaptopyruvate (3-MP) by transferring its amines to α-ketoglutarate catalyzed by cysteine aminotransferase (CAT). 3-Mercaptopyruvate thiotransferase (3-MST) catalyzes the sulfur of 3-MP to convert into H_2_S. In cardiomyocytes that mainly express CSE, H_2_S is produced with L-cysteine as substrate under the catalysis of CSE. (The part in the figure is marked in red.) H_2_S plays important roles in many physiological processes, including vasodilation, blood pressure reduction, anti-apoptosis, anti-inflammation, anti-oxidative stress, cell survival/death, cell differentiation, cell proliferation/hypertrophy, mitochondrial bioenergetics/biogenesis, and endoplasmic reticulum stress.

For many years, H_2_S has been considered to be a toxic and odorous gas. However, since the 1990s, many studies have shown that H_2_S, along with NO and CO, belongs to the category of gasotransmitters (Wang et al., 2020b). Three “classic” H_2_S-producing enzymes have been identified: cystathionine-γ-lyase (CSE), cystathionine-β-synthase (CBS), and 3-mercaptopyruvate thiotransferase (3-MST) (Rose et al., 2017). The expression of H_2_S-producing enzyme is subcellular and tissue-specific. At the cellular level, CSE occurs strictly in the cytoplasm, while cysteine aminotransferase (CAT) is located in the mitochondria. In terms of tissue specificity, CSE is the most abundant in the cardiovascular system, while CBS is dominant in the nervous system and liver and is expressed in the heart (Kar et al., 2019; Shen et al., 2019). In the process of endogenous H_2_S production, CBS catalyzes the β-substitution reaction of homocysteine with serine to produce L-cystathionine. L-cystenine is produced by the elimination of α, γ-cysteine of L-cystathionine catalyzed by CSE. L-cystenine then produces H_2_S via β elimination reaction catalyzed by CSE/CBS. L-cystenine also produces 3-mercaptopyruvate (3-MP) by transferring its amines to α-ketoglutarate catalyzed by CAT. 3-MST catalyzes the sulfur of 3-MP to convert into H_2_S. In cardiomyocytes that mainly express CSE, H_2_S is produced with L-cysteine as substrate under the catalysis of CSE (Figure 1) (Behera et al., 2019). In addition, there are some other recognized or assumed sources of H_2_S, including D-amino acid oxylase (Han et al., 2020) and methionine oxidase (Pol et al., 2018). In biological systems, several non-enzymatic methods can also produce H_2_S (Yang et al., 2019). In T2DM patients with DCM, endogenous H_2_S production by CSE is inhibited in cardiomyocytes (Mard et al., 2016). H_2_S level in plasma is decreased, and the supplement of H_2_S can reduce the cardiomyopathy dysfunction induced by hyperglycemia (Kar et al., 2019).

H_2_S can act as a signal molecule immediately after it is released, and it can also be stored as bound endosulfan, which can release H_2_S. At physiological pH, nearly two-thirds of H_2_S is in the form of hydrogen sulfide anion (HS–) (Guo et al., 2016). H_2_S plays important roles in many physiological processes, including vasodilation, blood pressure reduction (Greaney et al., 2017; Jin et al., 2017), anti-apoptosis (Li et al., 2019b), anti-inflammation (Zhao et al., 2019), anti-oxidative stress (Tocmo and Parkin, 2019), cell survival/death, cell differentiation, cell proliferation/hypertrophy, mitochondrial bioenergetics/biogenesis, and endoplasmic reticulum stress (ERS) (Figure 1) (Zhang D. et al., 2017). Recent studies have shown that H_2_S ameliorates diabetic complications including endothelial dysfunction (Li et al., 2019a), nephropathy (Karmin and Siow, 2018), retinopathy (Wang P. et al., 2019), and cardiovascular diseases (Citi et al., 2018). Research has reported that in diabetes, intraislet H_2_S could promote opening of the ATP dependent potassium channel, increase K^+^ efflux to lead to cell membrane hyperpolarization, and then close the L-type voltage-dependent calcium channel, thus inhibiting insulin secretion of pancreatic beta cells. On the contrary, much literature has shown that H_2_S can promote the release of insulin from β-cells. The reason for the above contradiction has not been fully studied (Szabo, 2012; Piragine and Calderone, 2020). In addition, H_2_S could also regulate the apoptosis of islet beta cells and increase ERS and apoptosis of pancreatic beta cells by inhibiting the extracellular signal regulated kinase (ERK) and activating p38 mitogen activated protein kinase (p38 MAPK) signal pathway (Yang et al., 2007). Other studies have shown that H_2_S could inhibit high glucose (HG)–induced apoptosis of pancreatic beta cells through the antioxidant, anti-inflammatory, or protein kinase B (Akt) signaling pathways (Taniguchi et al., 2011). However, the mechanism of H_2_S in diabetes is not fully understood.

## The Role of H_2_S in DCM

### Exogenous H_2_S Improves DCM by Inhibiting Oxidative Stress, Inflammation, and Apoptosis

One of the important pathophysiological factors of DCM is that persistent hyperglycemia induces oxidative stress and apoptosis to lead to cardiac fibrosis (Boudina and Abel, 2007). It has been reported that blocking the formation of reactive oxygen species (ROS) inhibited the expression of the fibrosis-related factor matrix metalloproteinase-2 (MMP-2) induced by HG (Yamagishi et al., 2001). Exogenous H_2_S can suppress oxidative stress by inhibiting ROS production (Xiao et al., 2018), which may be the mechanism of its anti-diabetic myocardial fibrosis. Nuclear factor kappa-B (NF-κB) is a ubiquitous inducible transcription factor. Activation of NF-κB can upregulate the expression of fibrosis-related factors, including transforming growth factor b1 (TGF-b1) and MMP-2, and increase Fas-ligand (Fas-L) expression to induce Fas-L–mediated apoptosis in the myocardium of diabetic rats, which leads to myocardial fibrosis (Darville and Eizirik, 2001; Chen et al., 2003; Hein et al., 2003). NaHS (a donor of H_2_S) treatment may play an anti-fibrogenic role by reducing HG-induced expression of NF-κB and the fibrogenic factor such as TGF-b1 and MMP-2 to improve diabetic myocardial fibrosis (El-Seweidy et al., 2011). Therefore, it can be inferred that H_2_S ameliorates DCM by inhibiting oxidative stress and apoptosis through suppressing the NF-κB pathway and ROS production.

Except for oxidative stress and apoptosis, inflammation is involved in the pathogenesis of DCM (Al-Rasheed et al., 2017; Zhao et al., 2017; Zou et al., 2019). A chronic, low-level state of systemic and sterile inflammation is an important feature of DCM (Sharma et al., 2018). The NLRP3 inflammasome is an important complex protein that mediates inflammation (Wang H. et al., 2019). Studies showed that suppressing NLRP3 notably improved DCM (Ye et al., 2017). H_2_S decreases HG-induced cell apoptosis, ROS production, and the expression of the NLRP3 inflammasome, Toll-like receptor 4 (TLR4), and NF-κB. NLRP3 gene silencing improves HG-induced cell apoptosis and inflammation, and the TLR4/NF-κB pathway mediates the activation of NLRP3 induced by HG in cardiac cells. These results indicate that H_2_S improves DCM by inhibiting HG-induced cardiomyocyte inflammation and apoptosis by suppressing the NLRP3 inflammasome through inhibiting the TLR4/NF-κB pathway (Huang et al., 2016). The NLRP3 inflammasome will be an important target for H_2_S to improve DCM. LV dysfunction, myocardial hypertrophy, and myocardial fibrosis are important pathological changes of DCM. H_2_S can improve LV function and inhibit myocardial hypertrophy and myocardial fibrosis to ameliorate DCM by suppressing inflammation, oxidative stress, and apoptosis induced by HG. Mechanism studies show that H_2_S suppresses HG-induced oxidative stress by activating the nuclear factor erythroid 2–related factor 2(Nrf2)/antioxidant response element (ARE) pathway, decreases HG-induced apoptosis through inhibiting the c-Jun N-terminal kinase (JNK)/p38 MAPK pathways, and activating the phosphatidylinositol 3-kinase (PI3K)/Akt pathway (Zhou et al., 2015). Another pathway, the AMPK/mTOR pathway, is also involved with the H_2_S protection of H9c2 cells against HG-induced apoptosis. GYY4137 (a donor of H_2_S) treatment improves HG-induced cell viability decrement. Moreover, both GYY4137 and AICAR (an AMPK activator) increase AMPK phosphorylation, decrease mammalian target of rapamycin (mTOR) phosphorylation, and ameliorate HG-induced cell viability decrement. Moreover, AraA (an AMPK inhibitor) attenuates the cardioprotection of GYY4137. Collectively, it can be inferred that exogenous H_2_S improves DCM through suppressing HG-induced apoptosis by activation of the AMPK/mTOR signal pathway (Wei et al., 2014).

Forkhead box protein O1 (FoxO1), which is a member of the FoxO family, plays a significant role in DCM through affecting oxidative stress, metabolism, inflammation, and apoptosis (Chistiakov et al., 2017). Exogenous H_2_S can ameliorate cardiac function, myocardial fibrosis, and hypertrophy in diabetic mice by inhibiting oxidative stress and apoptosis induced by HG. Mechanism studies show that H_2_S promotes the phosphorylation level of FoxO1 and suppresses FoxO1 nuclear translocation in cardiomyocytes via S-sulfhydration, which inhibits apoptosis and suppresses oxidative stress by increasing the expression of antioxidant enzymes. Therefore, it can be inferred that exogenous H_2_S ameliorates DCM via the FoxO1 pathway (Ye et al., 2018). FoxO1 may be an important target for H_2_S to improve DCM. The Wnt/β-catenin pathway also plays an important role in H_2_S protection of DCM. Zhang and Ye found that H_2_S could reduce the levels of ROS and malondialdehyde and increase the activities of superoxide dismutase, catalase, and glutathione peroxidase to attenuate HG-induced cardiomyocyte apoptosis and oxidative stress by suppressing the Wnt/β-catenin pathway, which need to be further studied, especially the relationship between the Wnt/β-catenin pathway and apoptosis (Zhang and Ye, 2019).

AGEs are proteins or lipids that become glycosylated after exposure to sugars. The receptor of advanced glycated product (RAGE) is a transmembrane receptor of the immunoglobulin superfamily, which can lead to oxidative stress, inflammation, and apoptosis. It has been found that H_2_S can inhibit oxidative stress, inflammation, and apoptosis induced by RAGE over-activation through inhibiting RAGE dimer formation and impairing its protein stability (Zhou et al., 2017). Whether RAGE mediates the protective effect of H_2_S on the diabetic myocardium is worth discussing. The signal transduction pathways involved in the above processes are summarized in Table 1.

**Table 1:** The signaling pathways involved in exogenous hydrogen sulfide (H_2_S) improvements of diabetic cardiomyopathy by inhibiting oxidative stress, inflammation, and apoptosis.

**Signaling pathway**	**The mode of action of H_**2**_S on signal pathway**	**Effects**
NF-κB pathway	Inhibition	Suppressing oxidative stress
TLR4/ NF-κB pathway	Inhibition	Suppressing inflammation and apoptosis
Nrf2/ARE pathway	Activation	Suppressing oxidative stress
NK/p38 MAPK pathway	Inhibition	Suppressing apoptosis
PI3K/Akt pathway	Activation	Suppressing apoptosis
AMPK/mTOR pathway	Activation	Suppressing apoptosis
FoxO1 pathway	Phosphorylation	Suppressing oxidative stress and apoptosis
Wnt/ B-catenin pathway	Inhibition	Suppressing oxidative stress and apoptosis

*NF-κB, nuclear factor kappa-B; TLR4, Toll-like receptor 4; Nrf2, nuclear factor erythroid 2–related factor 2; ARE, antioxidant response element; JNK, c-Jun N-terminal kinase; p38 MAPK, p38 mitogen activated protein kinase; PI3K, phosphatidylinositol 3-kinase; mTOR, mammalian target of rapamycin; FoxO1, Forkhead box protein O1*.

### Exogenous H_2_S Improves DCM by Regulating ERS

It has been reported that ERS can be induced in the diabetic heart and participate in the pathogenesis of DCM (Lian et al., 2017). Cardiac lipid toxicity refers to the direct toxic effect of excessive lipid deposition on myocardial cell function, which may be an important part of the phenotype of DCM. Both exogenous H_2_S and ERS inhibitors (4-PBA) can improve DCM by decreasing palmitic acid (PA)–induced myocardial injury. Similar results can be obtained in diabetic rats by using NaHS or 4-PBA. In addition, exogenous H_2_S inhibits ERS in diabetic rats or PA-induced AC 16 cardiac cells by decreasing the expressions of CHOP, GRP78, and caspase-12; therefore, it can be inferred that exogenous H_2_S can ameliorate DCM by inhibiting ERS (Guo et al., 2017). The relationship between ERS and apoptosis needs to be clarified, and how H_2_S regulates lipid metabolism through ERS deserves further study. It has been reported that ROS overproduction in DCM is often accompanied by ERS and that ROS/ERS-mediated apoptosis is involved in the pathogenesis and development of DCM. Exogenous H_2_S can improve the myocardial injury of diabetic rats, reduce the expression of ERS-related proteins, and improve the injury of H9c2 cells induced by HG. In HG-induced H9C2 cells, exogenous H_2_S notably reduced intracellular ROS levels, while an ROS scavenger decreases HG-induced apoptosis, indicating that ROS-induced cardiomyocyte apoptosis mediates the protective effect of exogenous H_2_S on the diabetic myocardium. HG can induce ROS and ERS, while an ROS scavenger can inhibit the expression of ERS-related proteins induced by HG, suggesting that HG increases ROS level to promote ERS. In addition, in an HG-induced H9C2 cell, HG induces Mfn-2 expression, and siRNA targeting Mfn-2 reduces ROS-induced apoptosis and HG-induced ERS, indicating that HG increases ROS/ERS-mediated apoptosis via Mfn-2. Based on the above, it can be inferred that exogenous H_2_S ameliorates DCM by decreasing ROS/ERS-mediated apoptosis through suppression of Mfn-2 expression (Yang et al., 2017). The relationship of ERS and Mfn-2 requires further study.

### Exogenous H_2_S Improves DCM by Regulating Autophagy

Type 2 diabetes is characterized by protein misfolding and aggregation, which results in mitochondrial damage, ROS production, and apoptosis and leads to ubiquitin aggregates (Chiti and Dobson, 2006; Shang and Taylor, 2011; Sato et al., 2014). Ubiquitin aggregation, which also leads to apoptosis and ROS production, is mainly eliminated by autophagy (Zhang Y. et al., 2017; Grumati and Dikic, 2018). Autophagy plays a protective role in DCM by clearing misfolded protein and ubiquitin (Pei et al., 2018). In diabetic mice, exogenous H_2_S can ameliorate DCM by decreasing ROS production. Exogenous H_2_S also can promote the degradation of autophagosome content, decrease the expression of p62, and increase the expression of microtubule associated protein 1 light chain 3 II (LC3II), autophagy associated protein 7 (Atg7), and Beclin1, indicating that exogenous H_2_S promotes autophagy. Moreover, exogenous H_2_S increases the expression of kelch-like ECH related protein 1 (keap-1) by decreasing its ubiquitylation, and Keap-1 siRNA inhibits the effect of exogenous H_2_S on autophagy in the cardiomyocyte of diabetic rats, indicating that exogenous H_2_S promotes autophagy through keap-1. Further research shows that 1,4-dithiothreitol, an inhibitor of disulfide bonds, counteracts the effects of H_2_S on keap-1, ubiquitin aggregates clearance and ROS production in HG-induced H9C2 cells, and H_2_S can promote the formation of disulfide between two keap-1 molecules, which indicates that exogenous H_2_S suppresses Keap-1 ubiquitylation through promoting its disulfide formation. From the above results, it can be inferred that exogenous H_2_S improves DCM by promoting ubiquitin aggregation clearance through promoting autophagy via ubiquitylation of Keap-1, which contributes to ROS scavenging and provides a new mechanism for the antioxidation of H_2_S. In addition, exogenous H_2_S has no notable effect on Nrf2 nuclear translocation, indicating that its antioxidant effect is not related with the keap-1/Nrf2 signaling pathway (Wu et al., 2017). The mechanism of exogenous H_2_S acting as an antioxidant through autophagy needs further study.

### Exogenous H_2_S Improves DCM by Improving Cardiac Mitochondrial Function

Recent evidence suggests that DCM is associated with metabolic abnormalities, more often with mitochondrial dysfunction. Sirtuin 3 (SIRT3) belongs to the nicotinamide adenine dinucleotide (NAD)–dependent deacetylase family and is the major mitochondrial deacetylase of lysine residues. In cardiac mitochondria of diabetic mice, cardiac mitochondrial respiratory capacities, ATP synthesis, and the expression and activity of SIRT3 were decreased, and exogenous H_2_S increased the expression and activity of SIRT3 by restoring the ratio of NAD^+^/NADH, and decreased the acetylation levels of the mitochondrial respiratory complex enzymes to improve cardiac mitochondrial dysfunction. SiRNA-mediated SIRT3 silencing increases the acetylation level of mitochondrial respiratory complexes, while exogenous H_2_S partially restores the acetylation level of these enzymes. Collectively, it can be inferred that exogenous H_2_S improves DCM by improving cardiac mitochondrial function through increasing the expression of SIRT3 to regulate the lysine acetylation of mitochondrial respiratory complexes (Sun Y. et al., 2019). The role of SIRT3 in H_2_S improving DCM remains to be further studied.

### Exogenous H_2_S Improves DCM by Activating K_ATP_ Channels

The K_ATP_ channels are abundant in the myocardium (Nichols and Lederer, 1991). The opening of K_ATP_ channels can reduce the apoptosis induced by oxidative stress in cardiac cells to improve DCM (Akao et al., 2001). Liang et al. found that the expression levels of K_ATP_ channels were decreased by HG, which was abolished by exogenous H_2_S. HG or K_ATP_ channel blocker could induce in H9c2 cardiac cells considerable injuries, including reducing cell viability, increasing apoptosis, ROS generation, and cleaved caspase-3 expression, as well as the loss of MMP. However, exogenous H_2_S or K_ATP_ channel openers could reverse the changes. Collectively, it could be inferred that exogenous H_2_S could improve DCM by activating K_ATP_ channels. Moreover, an ROS scavenger could ameliorate the reduction in the expression levels of the K_ATP_ channel induced by HG, and K_ATP_ channel openers could decrease HG-induced ROS production, indicating that there is an interaction between K_ATP_ channels and ROS and that the interaction is involved in the above H_2_S improvement of DCM (Liang et al., 2016). The role of K_ATP_ channels in the improvement of DCM by H_2_S, especially the interaction between K_ATP_ channels and ROS, needs to be further studied. K_ATP_ channels will become an important target for H_2_S to improve DCM.

### The Roles of Endogenous H_2_S in DCM

In addition to exogenous H_2_S, endogenous H_2_S can also improve DCM. El-Sayed et al. found that HG decreased CSE expression/activity, H_2_S, and serum adiponectin level and increased myocardial imidazoline I_1_ receptor expression, while moxonidine (imidazoline I_1_ receptor agonist) abolished the above effects of HG and improved the glycemic state; reversed myocardial hypertrophy, hypertension, and baroreflex dysfunction in streptozocin (STZ)-treated rats; and inhibited the expression of death associated protein kinase-3 (DAPK-3) to play **c**ardiovascular protective effects in diabetic mice. Moreover, inhibition of CSE decreased endogenous H_2_S production and counteracted moxonidine protective effects, indicating that CSE-derived H_2_S might mediate the cardiovascular protective effects of moxonidine in diabetes (El-Sayed et al., 2016). So far, there are few studies on moxonidine and endogenous H_2_S, so how moxonidine promotes the production of CSE-derived H_2_S remains to be further explored.

## Conclusion

**Figure 2:**
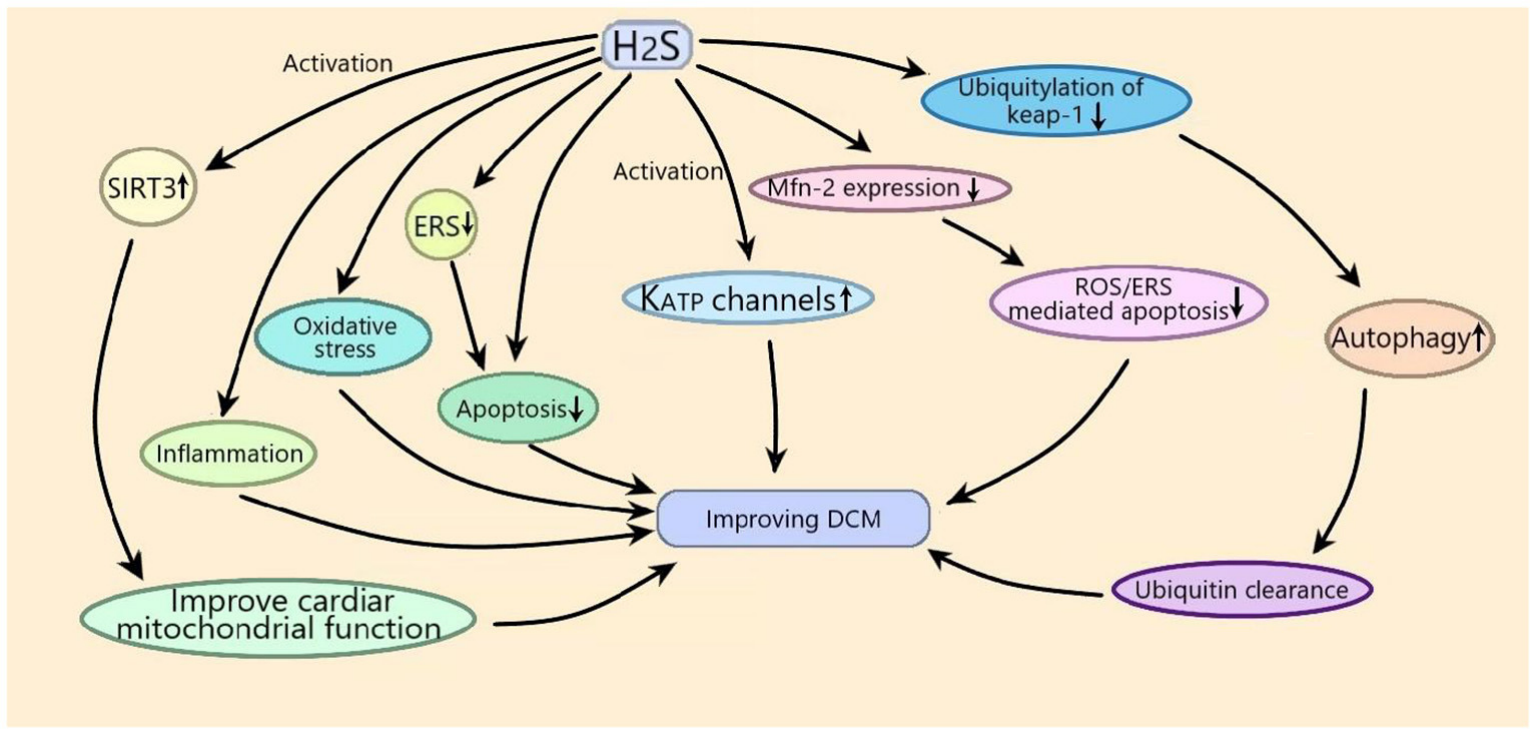
Schematic diagram of the mechanism of exogenous H_2_S improving diabetic cardiomyopathy (DCM). Exogenous H_2_S improves DCM by suppressing oxidative stress, inflammation, and apoptosis. Exogenous H_2_S improves DCM by inhibiting apoptosis through suppressing endoplasmic reticulum stress (ERS) or inhibiting reactive oxygen species (ROS)/ERS–mediated apoptosis through suppressing Mfn-2 expression. Exogenous H_2_S improves DCM by suppressing ubiquitylation of kelch-like ECH related protein 1 (Keap-1) to promote autophagy for ubiquitin clearance. Exogenous H_2_S improves DCM by improving cardiac mitochondrial function through activating sirtuin 3 (SIRT3). Exogenous H_2_S could improve DCM by activating K_ATP_ channels.

Exogenous H_2_S can improve DCM by suppressing oxidative stress, inflammation, and apoptosis. Exogenous H_2_S improves DCM by inhibiting apoptosis through suppressing ERS or inhibiting ROS/ERS-mediated apoptosis through suppressing Mfn-2 expression. Exogenous H_2_S improves DCM by suppressing ubiquitylation of Keap-1 to promote autophagy for ubiquitin clearance. Exogenous H_2_S improves DCM by improving cardiac mitochondrial function through activating SIRT3. Exogenous H_2_S could improve DCM by activating K_ATP_ channels (Figure 2). It can be seen from the above that the anti-inflammatory, anti-apoptotic, and antioxidant effects of H_2_S have potential therapeutic value in DCM, but its mechanism has not been fully studied, especially the signal transduction pathways involved. Autophagy, the NLRP3 inflammasome, and ERS are the regulated targets of H_2_S and are involved in the process of improving DCM by H_2_S, so the interaction among the three in H_2_S improvement of DCM should be studied in the future. ERS and NLRP3 inflammasome crosstalk has been reported to play an important role in metabolic disorders (Ji et al., 2019), and H_2_S regulates the ERS/NLRP3 inflammasome in many diseases (Wang et al., 2020a,b), so whether H_2_S can regulate the ERS/NLRP3 inflammasome to improve diabetes deserves to be studied. In addition, the existing H_2_S releasers cannot fully meet the requirements of research and development of H_2_S related drugs, so the development of new H_2_S releasers is very important for the application of H_2_S related drugs in the treatment of clinical diseases.

**Figure 3:**
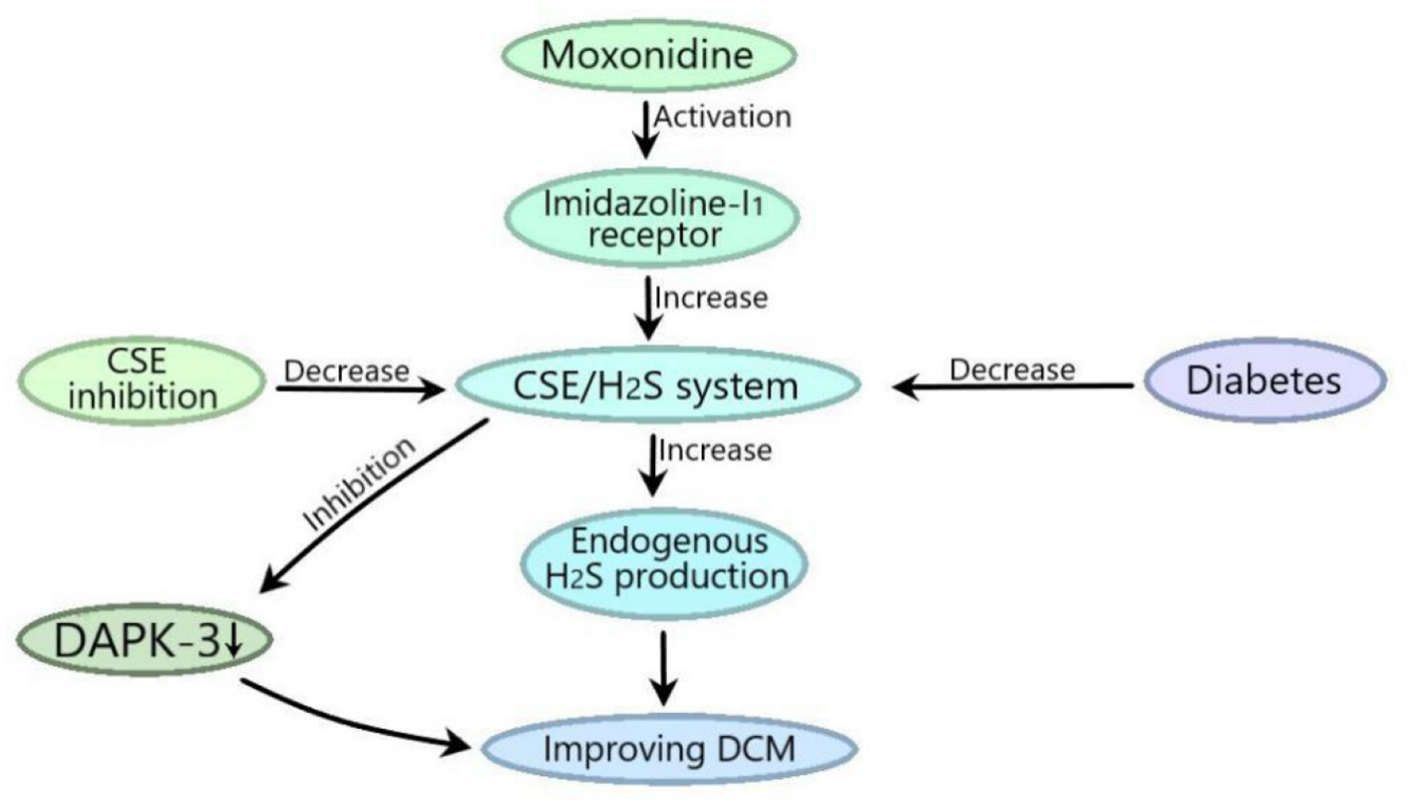
CSE-derived H_2_S mediates the moxonidine improvements of DCM. Diabetic state can inhibit the CSE/H_2_S system, and moxonidine can activate myocardial imidazoline I1 receptor to improve DCM by promoting the CSE/H_2_S system to increase endogenous H_2_S production or by promoting the CSE/H_2_S system to inhibit death related protein kinase 3 (DAPK-3).

Diabetic states can inhibit endogenous H_2_S-producing enzyme CSE, while the activation of myocardial imidazoline I1 receptor with moxonidine can improve DCM by increasing endogenous H_2_S via CSE or increase CSE expression to inhibit DAPK-3 to ameliorate DCM (Figure 3). Myocardial imidazoline I1 receptor is an important target for the development of therapeutic drugs for DCM.

In conclusion, with further research, H_2_S could provide new ways of treating DCM.

## Author Contributions

HW: devised, writing, and funded with this review. XiaL: drawing. SZ: writing and funded with this review. XW: writing. All authors contributed to the article and approved the submitted version.

## Conflict of Interest

The authors declare that the research was conducted in the absence of any commercial or financial relationships that could be construed as a potential conflict of interest.
